# Neoplastic cell percentage estimation in tissue samples for molecular oncology: recommendations from a modified Delphi study

**DOI:** 10.1111/his.13891

**Published:** 2019-07-18

**Authors:** Kelly Dufraing, J Henricus van Krieken, Gert De Hertogh, Gerald Hoefler, Anca Oniscu, Tine P Kuhlmann, Wilko Weichert, Caterina Marchiò, Ari Ristimäki, Aleš Ryška, Jean‐Yves Scoazec, Elisabeth Dequeker

**Affiliations:** ^1^ Biomedical Quality Assurance Research Unit, Department of Public Health and Primary Care KU Leuven Leuven Belgium; ^2^ Department of Pathology Radboud University Medical Center Nijmegen the Netherlands; ^3^ Department of Pathology University Hospital Leuven Leuven Belgium; ^4^ Diagnostic and Research Institute of Pathology Medical University of Graz Graz Austria; ^5^ Department of Molecular Pathology, Laboratory Medicine Royal Infirmary of Edinburgh Edinburgh UK; ^6^ Department of Pathology Herlev Hospital Copenhagen Denmark; ^7^ Department of Pathology Technical University Munich Munich Germany; ^8^ Department of Medical Sciences University of Turin and Pathology Unit Torino Italy; ^9^ FPO‐IRCCS Candiolo Cancer Institute Candiolo Italy; ^10^ Department of Pathology, Research Programs Unit and HUSLAB University of Helsinki and Helsinki University Hospital Helsinki Finland; ^11^ The Fingerland Department of Pathology Faculty of Medicine and University Hospital Hradec Kralove Czech Republic; ^12^ Pathology Department Gustave Roussy Villejuif France

**Keywords:** molecular biomarker testing, neoplastic cell percentage, recommendations

## Abstract

**Aims:**

Results from external quality assessment revealed considerable variation in neoplastic cell percentages (NCP) estimation in samples for biomarker testing. As molecular biology tests require a minimal NCP, overestimations may lead to false negative test results. We aimed to develop recommendations to improve the NCP determination in a prototypical entity – colorectal carcinoma – that can be adapted for other cancer types.

**Methods and results:**

A modified Delphi study was conducted to reach consensus by 10 pathologists from 10 countries with experience in determining the NCP for colorectal adenocarcinoma. This study included two online surveys and a decision‐making meeting. Consensus was defined a priori as an agreement of > 80%. All pathologists completed both surveys. Consensus was reached for 8 out of 19 and 2 out of 13 questions in the first and second surveys, respectively. Remaining issues were resolved during the meeting. Twenty‐four recommendations were formulated. Major recommendations resulted as follows: only pathologists should conduct the morphological evaluation; nevertheless molecular biologists/technicians may estimate the NCP, if specific training has been performed and a pathologist is available for feedback. The estimation should be determined in the area with the highest density of viable neoplastic cells and lowest density of inflammatory cells. Other recommendations concerned: the determination protocol itself, needs for micro‐ and macro‐dissection, reporting and interpreting, referral practices and applicability to other cancer types.

**Conclusion:**

We believe these recommendations may lead to more accurate NCP estimates, ensuring the correct interpretation of test results, and might help in validating digital algorithms in the future.

## Introduction

Molecular analysis of solid tumours has become standard practice for many cancer types. Because the outcome of those tests has a significant impact on deciding which systemic therapy is used in the individual patient, the testing process must be as precise and reliable as possible.^1^, ^2^ Much has been written about avoiding errors related to certain test methodologies.^3^, ^4^, ^5^, ^6^ Before tumour DNA is analysed, the most suitable tissue block must be selected. Important aspects of the pre‐analytical process include identification of the tumour area, determination of the neoplastic cell percentage (NCP) within that area, verification of the representativeness of the sample and, finally, the diagnosis, especially in situations when the tumour tested represents a metastasis. As testing strategies require a minimal amount of neoplastic cells in the samples tested for a reliable molecular test result (a parameter which directly influences the sensitivity of the test), it is important to select a sample that contains a sufficient number and/or percentage of neoplastic cells.^7^ Most molecular testing techniques require at least 5–10 ng DNA for a reliable analysis, which generally correspond to a minimum of 850–1700 cells.^8^, ^9^ Test failures in routine testing or during clinical trials have indeed been reported due to an insufficient NCP.^10^, ^11^, ^12^, ^13^ The NCP is also key for correct interpretation of the test outcome. Testing samples with insufficient NCPs might lead to false negative results, as it is uncertain whether the sample truly does not contain a variant or whether there was an insufficient number of neoplastic cells. When next‐generation sequencing (NGS) is used, an accurate NCP determination is essential to distinguish signals from noise, as the variant allelic frequency (VAF) can only be interpreted in the context of the NCP.^14^ In this scenario, if the NCP is overestimated, it might also be mistakenly thought that a tumour subclone is present. NCPs also have an impact on the interpretation of NGS quality metrics, as samples with low NPCs require higher coverages.^5^, ^15^ In addition, correct NCP estimation aids in distinguishing somatic from germline variants, with implications for the management of the patient and their relatives.

Several studies have already shown significant variation in NCP estimates; on average, as high as 20% between different pathologists.^16^, ^17^, ^18^ This was recently confirmed by a large European‐wide study, which also identified large differences in the processes related to the estimation of the NCP, such as contouring the tumour area and macrodissection.^19^ In addition, this study identified problems with interpreting the NCP. Here we address these issues and provide consensus‐based recommendations for the determination of the NCP and related processes as a first step to decrease interobserver variation and to increase the awareness of the importance of correct NCP determination.

## Methods

To obtain consensus‐based recommendations, a modified version of the Delphi method was used.^20^ The study consisted of two surveys and two conferences. This study was conducted for colorectal adenocarcinoma in view of the high frequency and importance of molecular biomarker testing in this cancer type.

### Expert Selection

To assure credibility with the target audience, 10 experienced pathologists from 10 different countries (Austria, Belgium, Czech Republic, Denmark, Finland, France, Germany, Italy, the Netherlands and United Kingdom) were selected to participate based on previous experience in molecular pathology, relevant publications and collaborations.

### Delphi Rounds

For the first survey, statements regarding the determination of the neoplastic cell content were drafted based on literature review and a survey that was previously conducted as part of an external quality assessment scheme for colorectal cancer, as described elsewhere.^19^ The expert panel was asked to indicate the extent to which they agree with the statements and to explain their choice. Consensus was defined a priori as an agreement of > 80% between the experts. Statements for which no consensus was reached were formulated again in the second survey, together with an overview of the different views. Furthermore, the experts were given the opportunity to include additional statements during the first survey.

In round 3, the experts met personally for a 2‐day meeting aimed at achieving a final consensus. Each statement was discussed with a focus on those for which no consensus had been reached. After a short discussion, a final vote was held. Afterwards, the newly proposed protocol for the selection of the area in which the NCP should be determined and the estimation itself were tested in practice using a multihead microscope on 25 haematoxylin and eosin (H&E)‐stained slides from formalin‐fixed paraffin‐embedded (FFPE) resection specimens from colorectal adenocarcinoma.

After the meeting, the revised consensus statements were distributed to the experts present at the meeting to obtain their agreement on the exact wording of the statements. Subsequently, the statements were forwarded to the pathologists who had not been present at the onsite meeting. Final consensus was obtained afterwards during a teleconference.

## Results

All invited pathologists agreed to participate as experts in this Delphi study, except for one pathologist who suggested a colleague with comparable experience. The experts had, on average, 12 years of experience in molecular pathology with a routine evaluation of at least 10 slides per week during their diagnostic practice.

The first survey contained 19 statements subdivided into the categories described below. Two statements were considered improperly worded, and consensus was reached on eight statements. The second survey included the 11 remaining statements for which no consensus had been originally reached, including the different opinions and two additional statements that had emerged from expert feedback. During the consensus meeting 24 recommendations regarding the determination of the NCP were formulated and categorised (Table 1).

**Table 1:** Consensus‐based recommendations related to estimating the neoplastic cell percentage before molecular analysis

Part 1: General statements	
1.	The neoplastic cell percentages (NCP) should be determined in daily practice
2.	Every case must be evaluated (morphological evaluation and diagnosis) by a pathologist before a molecular test is performed
3.	The estimation of the NCP should be performed by the pathologist evaluating the case, but may also be performed by a molecular biologist/technician who attended a training programme that is officially recognised by a national society, if available, and who has access to a pathologist for double checking, especially whenever encountering any unusual findings or difficult cases
Note	After molecular analysis, the haematoxylin and eosin (H&E) (on which the initial diagnosis was made) should be available for the pathologist who signs the molecular report
Part 2: Determination protocol	
4.	Manually counting individual cells is not suitable in daily practice
5.	The estimation should be made as accurately as possible in deciles
	Note 1: It is not sufficient to state ‘below or above the threshold’ Note 2: Categories might also be used as long as they are thoroughly validated and tuned between the different pathologists of one pathology department Note 3: Cases with NCPs close to the threshold should be discussed with the molecular biologist
6.	Gross estimation: percentage neoplastic cells versus all cells in a zone for dissection is most suitable
7.	Selection of the area for neoplastic cell content estimation: select the area with the highest density of viable neoplastic cells and the lowest density of inflammatory cells. Avoid areas of necrosis and mucus (Figure 1). It is better to have a smaller area with fewer non‐neoplastic cells than a larger area with many non‐neoplastic cells
8.	All non‐neoplastic nuclei (such as inflammatory cells, desmoplastic stroma and fat tissue) should be taken into account when estimating the neoplastic cell count
9.	All areas without nuclei (such as mucus and necrosis) can be visual confounders
10.	The tumour area can be marked by gross methods (such as gross circles and gross tumour shape), because this fits best with macrodissection Note: If there are only small areas with high neoplastic cell counts (for example, because of necrosis) or heterogeneous samples, indication by one or more small circles is also possible
11.	Suggested protocol for a surgical specimen (Figures 2 and 3): Select the most cellular area of the tumour (avoid artefacts) under low magnification Check at high magnification whether the area is homogeneous (inflammatory cell infiltrates, areas of necrosis, etc.) Contour the tumour area: ○ Homogeneous samples: Estimate the NCP at intermediate magnification as the percentage of neoplastic cells versus all cells in the area that will be used for macrodissection ○ Heterogeneous samples: Estimate multiple areas under intermediate magnification and take the average value Note: for endoscopic biopsies a high magnification should be used Take notes: ○ NCP ○ Conclusion: is the sample suitable or not for molecular downstream analyses?
13.	An H&E‐stained section cut after the molecular analysis may be analysed if required in the process, especially in endoscopic or image‐guided biopsies
Part 3: Need for micro‐ and macrodissection	
14.	Dissection should be performed in most cases, unless the whole section contains a sufficient NCP
15.	Macrodissection is generally sufficient, but depending on the sample a magnifier can be used Note: Macrodissection means ‘without the use of a microscope’
16.	Laser‐guided microdissection is not recommended for daily routine practice
17.	It is recommended to perform the dissection on unstained slides
18.	Whether the marked area corresponds to the macrodissected area in the process of macrodissection should be checked
Part 4: Reporting and interpretation	
19.	The NCP should be included in the report
20.	The NCP should be taken into account for interpretation of the test result
21.	The following warning note should be added when no mutation is identified and the NCP is below the threshold of the molecular technique: ‘Due to a low NCP, a false negative result cannot be excluded. If possible and clinically relevant, a specimen with higher NCP should be tested’
22.	The following warning note should be added for positive results when the NCP is below the threshold of the molecular technique, where appropriate: ‘Due to low neoplastic cell content, a false negative result for other tested genes cannot be excluded. If possible and relevant, a specimen with higher NCP should be tested’
Part 5: Additional items	
23.	When samples from external laboratories are to be analysed, the pathologist who receives the blocks from elsewhere and performs the molecular analysis should make the estimation or check if there is agreement with the original estimate, if any
24.	These recommendations are easy to apply and extend to other cancer types

**Figure 1:**
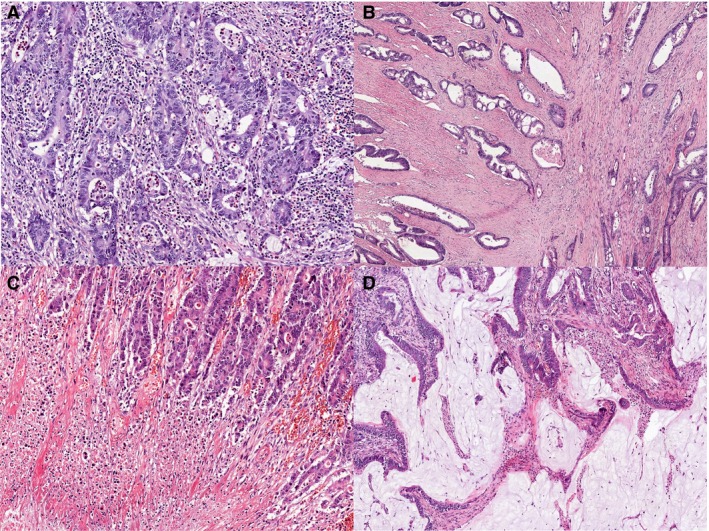
Examples of elements that should be avoided when contouring the tumour area. Images of haematoxylin and eosin‐stained formalin‐fixed paraffin‐embedded tissue from metastatic colorectal cancer showing examples of elements that should be avoided when contouring the tumour area. **A**, Abundant presence of inflammatory cells. **B**, Extensive presence of desmoplastic stroma. **C**, Abundant (‘dirty’) necrosis. **D**, High‐mucinous sample

**Figure 2:**
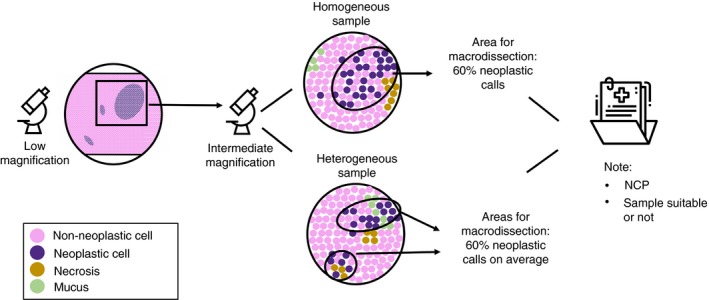
Proposed protocol for the determination of the neoplastic cell content. This figure visually illustrates the proposed protocol for estimating the neoplastic cell percentages (NCP). First, the most cellular area of the tumour has to be selected under low magnification and artefacts (as exemplified by Figure 1) should be avoided. At high magnification it should then be checked whether or not the area is homogeneous. For homogeneous samples the NCP could be estimated at intermediate magnification, as the percentage of neoplastic cells versus all cells in the area that will be used for macrodissection. In heterogeneous samples, the estimation is the average of estimates made under intermediate magnification in multiple areas. After determining the percentage neoplastic cells, the NCP should be noted, and also whether the sample is suitable for downstream analysis or not

**Figure 3:**
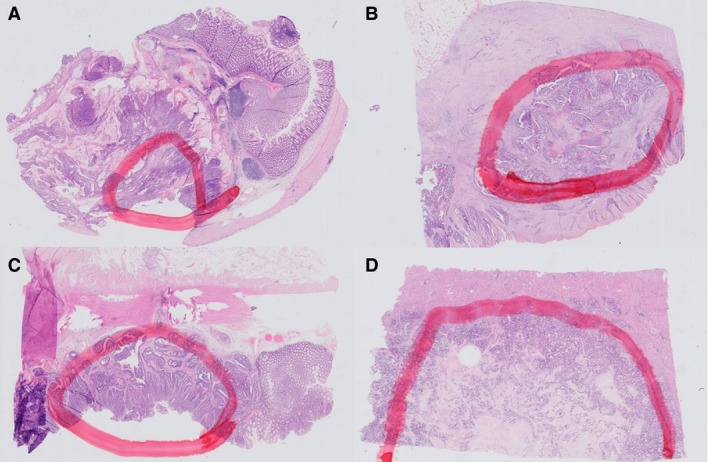
Examples of grossly delineated zones for microdissection. Images of haematoxylin and eosin‐stained formalin‐fixed paraffin‐embedded tissue from metastatic colorectal cancer with the gross delineation of the area to be macrodissected. **A**, Zone with 30% neoplastic cells. A mucinous zone was eliminated for macrodissection. **B**, Zone with 40% neoplastic cells, avoiding an area where solitary neoplastic cells are spread. **C**, Zone with 50% neoplastic cells. **D**, Zone with 70% neoplastic cells

Figure 1 shows examples of elements that should be avoided, such as infiltrating inflammatory cells, desmoplastic stroma, necrosis and mucus. Among inflammatory cells, only lymphocytes are easily visible on routine H&E stains, whereas macrophages can be quite easily underestimated. Finally, the experts want to stress that they prefer the term ‘neoplastic cells’ over ‘tumour cells’, as the tumour might also contain other cell types such as inflammatory cells.

## Discussion

Estimation of the NCP is regarded as a trivial step performed very quickly and routinely, yet each pathologist performs this differently and data published so far indicate that it is time to work on standardisation of this particular procedure. Accurate estimation is extremely important to correctly interpret the results of a molecular analysis which, in turn, is crucial for treatment decision‐making. Inaccurate estimations may impact upon patient outcomes.^19^ The present study proposes recommendations for standardising the processes related to defining the tumour area for macrodissection and for estimating the neoplastic cell percentage within this area.

As a previous study has already indicated, in most laboratories the pathologist determines the NCP.^19^ The expansion of available biomarkers has increased the workload of pathologists, and therefore the experts believe that the NCP could be estimated by molecular biologists or technicians, provided they are properly trained, and only after the tissue slide has been morphologically examined by the pathologist to confirm entity typing. ‘Proper training’ is defined by the experts as recognised training in molecular pathology organised by a national society. As this type of training is not available in most countries, an extensive internal training programme could be provided by the laboratory, as required by ISO 15189:2012 and by Cree *et al*.^21^, ^22^ The internal training must be properly supervised, followed by competence assessment and regular updates to maintain competency. Pathologists, as well as molecular biologists and technicians, could also consider attending external training sessions and benchmark themselves by comparing their estimates with colleagues or online training sets, if available. As the NCP is key to the accuracy of the downstream molecular analyses, experts believe that, on one hand, properly trained molecular biologists or technicians can assess this feature, and on the other hand a pathologist should always be available to double‐check and supervise the process, especially whenever unusual findings and/or difficult cases are encountered.

The estimation of the NCP should be performed grossly as the percentage neoplastic cells versus all cells in a zone for dissection. By ‘grossly’, we mean in contrast to counting individual cells or to counting individual cells in a limited area followed by multiplying this number for the total area. It is also important that the percentage of nuclei is estimated, and not the area the respective cells are covering. Large neoplastic cells are visually more outstanding than smaller lymphocytes, yet the amount of DNA in both cell types is comparable. It is recommended to make the estimation in deciles (e.g. 20%) or in categories which still enable correlation of the NCP with the variant allelic frequency (VAF) of the specified variant.^14^ For example, when a VAF is higher than expected, the variant might be a germline, or the gene might be affected by heterozygosity or tumour cell aneuploidy might have occurred. Conversely, if a low VAF is detected in a sample with a high NCP, the result could be interpreted as a testing artefact or as an indicator for subclonality of a given gene in the context of tumour heterogeneity. In these scenarios, testing an alternative tumour sample is good practice and helps to rule out artefacts. If categories are used, they should be thoroughly validated as part of the whole testing procedure and tuned between individual observers. Examples of categories are < 10, 10–30, 30–50, 50–90 and> 90%. Regardless of how the NCP is reported, it is critical to know the threshold (minimum required NCP) and sensitivity of the testing method. Estimates around this cut‐off should be made with extra care and discussed with the molecular laboratory where, when interpreting the quality metrics of the testing method, these can be acted upon.

When selecting the area with the highest density of viable neoplastic cells, areas with inflammatory cells, necrosis, desmoplastic stroma and mucus, as depicted in Figure 1, should be avoided, as those areas contain viable nuclei diluting the amount of neoplastic DNA. Although the study by Büttner *et al*. indicates that avoiding necrosis and mucus on colorectal specimen has no impact on the test outcome, others have set the acceptable amount of necrosis at 20% in various cancer types.^23^, ^24^, ^25^ It should be added that, when contouring the selected tumour area for macrodissection, a pen with an appropriate thickness should be used.

Macrodissection is always recommended, unless the sample contains a sufficient number (both absolute and relative) of viable neoplastic cells. Special attention should be paid when handling samples for molecular analysis to prevent contamination, as described elsewhere.^26^ Different methods are available for macrodissection, but no research has been conducted on their effects on test outcome. For example, tissue outside the contoured area can be scraped off using a diamond pen, or the contoured tissue can be scraped off and collected in a microtube.^26^, ^27^, ^28^ To increase dissection accuracy a magnifier can be used, but a microscope is not necessary according to the experts, as this is too time‐consuming. Given its high cost and complexity, laser‐guided microdissection is also not recommended to be used, apart from very rare cases where small areas or individual cells are essential to be analysed. With the current rapid development in digital pathology applications, other digitally guided dissection systems could enter laboratory practice during the next decade.^29^

For correct interpretation of the test result, it is important for the clinician to understand the test validity. Therefore, the NCP should be clearly included in the molecular test report, and the methodology section of the report should include test sensitivity and the list of variants that were included in the analysis. This is not only recommended by experts, but also by several international standards and guidelines.^21^, ^30^, ^31^, ^32^ When the NCP is below the threshold of the test method molecular testing should not be performed at all, or if the NCP is close to the threshold a disclaimer should be added to draw the clinician’s attention.^33^ This is not only recommended when no mutation is identified in the sample, but also for positive results in case of multiplex panel testing. Method sensitivities might differ per gene in the panel, and a positive result for one gene does not exclude a false negative result for another gene.

A modified form of the Delphi technique was used to obtain these consensus‐based recommendations. This technique has some major advantages, such as the ability to bring together experts from different countries, the structured communication process and time‐effectiveness. The technique also has a weakness, in that no guidelines exist for determining the level of consensus and the minimal number of experts needed.^34^ Therefore, we decided to set the consensus level in advance at an agreement of 80%, and we were of the opinion that the expertise and years of experience of the panel members is more important than the number of participants. Although this study focused on NCP processes for colorectal cancer, the experts believe that the proposed recommendations can be applied to other cancer and sample types (e.g. cytological preparations, including smears and cell blocks), taking into account the specific morphological properties of the tumour. One limitation related to cytological samples remains the reduced ability to macrodissect tumour out of cytological preparation due to the mixed cellularity often represented in these samples (‘contaminating’ inflammatory cells, mesothelial cells) and the somewhat high variation in cellularity between the levels analysed.

This study resulted in consensus‐based recommendations concerning practices related to the visual estimation of the NCP. Although the proposed protocols might not be adopted in the same way by all laboratories, this study also aimed to increase awareness of the importance of an accurate estimation of the NCP and its implications for high‐quality molecular testing, and eventually to reduce the variation in the practice of reporting the NCP. The possibilities of digital estimates also need to be further explored, as systems are becoming commercially available.

## Conflicts of interest

All authors have no conflicts of interest to declare.

## References

[1] Au TH , Wang K , Stenehjem D , Garrido‐Laguna I . Personalized and precision medicine: integrating genomics into treatment decisions in gastrointestinal malignancies. J. Gastrointest. Oncol. 2017; 8; 387–404. 28736627 10.21037/jgo.2017.01.04 PMC5506274

[2] VanderLaan PA , Rangachari D , Majid A *et al* Tumour biomarker testing in non‐small‐cell lung cancer: a decade of change. Lung Cancer 2018; 116; 90–95. 29413057 10.1016/j.lungcan.2018.01.002 PMC5806129

[3] Hiley CT , Le Quesne J , Santis G *et al* Challenges in molecular testing in non‐small‐cell lung cancer patients with advanced disease. Lancet 2016; 388; 1002–1011. 27598680 10.1016/S0140-6736(16)31340-X

[4] Sundar R , Chénard‐Poirier M , Collins DC , Yap TA . Imprecision in the era of precision medicine in non‐small cell lung cancer. Front. Med. 2017; 4; 39. 10.3389/fmed.2017.00039 PMC5385461 28443282

[5] Lin MT , Mosier SL , Thiess M *et al* Clinical validation of KRAS, BRAF, and EGFR mutation detection using next‐generation sequencing. Am. J. Clin. Pathol. 2014; 141; 856–866. 24838331 10.1309/AJCPMWGWGO34EGOD PMC4332779

[6] Lamy A , Blanchard F , Le Pessot F *et al* Metastatic colorectal cancer KRAS genotyping in routine practice: results and pitfalls. Mod. Pathol. 2011; 24; 1090–1100. 21516079 10.1038/modpathol.2011.60

[7] Loree JM , Kopetz S , Raghav KP . Current companion diagnostics in advanced colorectal cancer; getting a bigger and better piece of the pie. J. Gastrointest. Oncol. 2017; 8; 199–212. 28280626 10.21037/jgo.2017.01.01 PMC5334060

[8] Popper HH , Tímár J , Ryska A , Olszewski W . Minimal requirements for the molecular testing of lung cancer. Transl. Lung Cancer Res. 2014; 3; 301–304. 25806315 10.3978/j.issn.2218-6751.2014.10.02 PMC4367742

[9] Gillooly JF , Hein A , Damiani R . Nuclear DNA content varies with cell size across human cell types. Cold Spring Harb. Perspect. Biol. 2015; 7; a019091. 26134319 10.1101/cshperspect.a019091 PMC4484964

[10] Boissière‐Michot F , Lopez‐Crapez E , Frugier H *et al* KRAS genotyping in rectal adenocarcinoma specimens with low tumour cellularity after neoadjuvant treatment. Mod. Pathol. 2012; 25; 731–739. 22282307 10.1038/modpathol.2011.210

[11] Lewandowska MA , Jozwicki W , Zurawski B . KRAS and BRAF mutation analysis in colorectal adenocarcinoma specimens with a low percentage of tumour cells. Mol. Diagn. Ther. 2013; 17; 193–203. 23606169 10.1007/s40291-013-0025-8 PMC3663254

[12] Weichert W , Schewe C , Lehmann A *et al* KRAS genotyping of paraffin‐embedded colorectal cancer tissue in routine diagnostics: comparison of methods and impact of histology. J. Mol. Diagn. 2010; 12; 35–42. 20007841 10.2353/jmoldx.2010.090079 PMC2797716

[13] Kassahn KS , Holmes O , Nones K *et al* Point mutation calling in low cellularity tumours. PLoS ONE 2013; 8; 4380. 10.1371/journal.pone.0074380 PMC3826759 24250782

[14] Strom SP . Current practices and guidelines for clinical next‐generation sequencing oncology testing. Cancer Biol. Med. 2016; 13; 3–11. 27144058 10.28092/j.issn.2095-3941.2016.0004 PMC4850126

[15] Jennings LJ , Arcila ME , Corless C *et al* Guidelines for validation of next‐generation sequencing‐based oncology panels: a joint consensus recommendation of the Association for Molecular Pathology and College of American Pathologists. J. Mol. Diagn. 2017; 19; 341–365. 28341590 10.1016/j.jmoldx.2017.01.011 PMC6941185

[16] Lhermitte B , Egele C , Weingertner N *et al* Adequately defining tumour cell proportion in tissue samples for molecular testing improves interobserver reproducibility of its assessment. Virchows Arch. 2017; 470; 21–27. 27853865 10.1007/s00428-016-2042-6

[17] Smits AJ , Kummer JA , de Bruin PC *et al* The estimation of tumour cell percentage for molecular testing by pathologists is not accurate. Mod. Pathol. 2014; 27; 168–174. 23887293 10.1038/modpathol.2013.134

[18] Viray H , Li K , Long TA *et al* A prospective, multi‐institutional diagnostic trial to determine pathologist accuracy in estimation of percentage of malignant cells. Arch. Pathol. Lab. Med. 2013; 137; 1545–1549. 24168492 10.5858/arpa.2012-0561-CP

[19] Dufraing K , De Hertogh G , Tack V , Keppens C , Dequeker EMC , van Krieken HJH . External quality assessment identifies training needs to determine the neoplastic cell content for biomarker testing. J. Mol. Diagn. 2018; 20; 455–464. 29625250 10.1016/j.jmoldx.2018.03.003

[20] Powell C . The Delphi technique: myths and realities. J. Adv. Nurs. 2013; 41; 376–382. 10.1046/j.1365-2648.2003.02537.x 12581103

[21] International Organization for Standardization . ISO 15189: 2012 Medical laboratories – particular requirements for quality and competence. Geneva, Switzerland: ISO, 2012.

[22] Cree IA , Deans Z , Ligtenberg MJ *et al* Guidance for laboratories performing molecular pathology for cancer patients. J. Clin. Pathol. 2014; 67; 923–931. 25012948 10.1136/jclinpath-2014-202404 PMC4215286

[23] Büttner J , Lehmann A , Klauschen F *et al* Influence of mucinous and necrotic tissue in colorectal cancer samples on KRAS mutation analysis. Pathol. Res. Pract. 2017; 213; 606–611. 28551386 10.1016/j.prp.2017.04.028

[24] Cagle P , Allen TC , Beasley MB *et al* eds. Precision molecular pathology of lung cancer. Springer, NY: Molecular Pathology Library 2018; 79–86.

[25] Cancer Genome Atlas Research Network . Integrated genomic analyses of ovarian carcinoma. Nature 2011; 474; 609–615. 21720365 10.1038/nature10166 PMC3163504

[26] O’Grady A , Cummins R . Somatic DNA mutation analysis. Methods Mol. Biol. 2017; 1606; 219–233. 28502004 10.1007/978-1-4939-6990-6_15

[27] Marchetti I , Lessi F , Mazzanti CM *et al* A morphomolecular diagnosis of papillary thyroid carcinoma: BRAF V600E detection as an important tool in preoperative evaluation of fine‐needle aspirates. Thyroid 2009; 19; 837–842. 19534623 10.1089/thy.2009.0074

[28] Kotoula V , Charalambous E , Biesmans B *et al* Targeted KRAS mutation assessment on patient tumour histologic material in real time diagnostics. PLoS ONE 2009; 4; E7746. 19888477 10.1371/journal.pone.0007746 PMC2768905

[29] Geiersbach K , Adey N , Welker N *et al* Digitally guided microdissection aids somatic mutation detection in difficult to dissect tumours. Cancer Genet. 2016; 209; 42–49. 26767919 10.1016/j.cancergen.2015.12.004 PMC4738015

[30] Matthijs G , Souche E , Alders M *et al* Guidelines for diagnostic next generation sequencing. Eur. J. Hum. Genet. 2016; 24; 2–5. 26508566 10.1038/ejhg.2015.226 PMC4795226

[31] Richards S , Aziz N , Bale S *et al* Standards and guidelines for the interpretation of sequence variants: a joint consensus recommendation of the American College of Medical Genetics and Genomics and the Association for Molecular Pathology. Genet. Med. 2015; 17; 405–424. 25741868 10.1038/gim.2015.30 PMC4544753

[32] Organisation for Economic Cooperation and Development (OECD) . OECD guidelines for quality assurance in molecular genetic testing. Paris, France: OECD, 2007.

[33] Hébrant A , Froyen G , Maes B *et al* The Belgian next generation sequencing guidelines for haematological and solid tumours. Belg. J. Med. Oncol. 2017; 11; 56–67.

[34] Diamond IR , Grant RC , Feldman BM *et al* Defining consensus: a systematic review recommends methodologic criteria for reporting of Delphi studies. J. Clin. Epidemiol. 2014; 67; 401–409. 24581294 10.1016/j.jclinepi.2013.12.002

